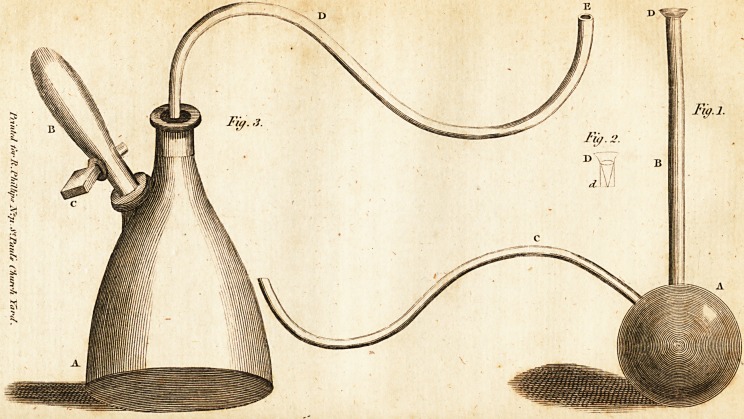# On the Means of Destroying the Virus of Ulcers, and Contagious Miasmata

**Published:** 1799-07

**Authors:** 


### *On the means of destroying the Virus of Ulcers, and Contagious Miasmata.*

[Extracted from Dr. Rollo's "Treatise on the *Diabetes Mellitus;*" and the "*Annales de Chemie*"; No. 86 and 87.]

In our preceding Number we gave a plate representing the apparatus for applying gases, or factitious air, to wounds, which was properly designed for the present Number; but, from accidental mistake of the engraver, as well as those who superintend the stitching, this plate has been misplaced. At present, therefore, we shall supply the explanation before omitted, and accompany it with a concise account of this improvement.

Cit. Guiton introduces this interesting memoir, in the 86th Number of the "Annales de Chimie," with the following liberal remark: "the manner in which Dr. Rollo treats this subject, makes it a branch of that extensive and hitherto unexplored science, animal chemistry; and affords us new hopes (as Dr. Rollo observes) of seeing the resources of medicine and surgery increased, by a practical application of the doctrine of pneumatic chemistry.

"Dr. Rollo examines the action of morbid matter on wounds. Independent of the ulcers which usually follow erysipelas, and of those which are known by the term of hospital ulcers, he believes he has observed a particular species proceeding from a deleterious germ which attaches to a part of the wound; which, like other poisons, possesses the property of *assimilation*, increasing by its own progressive virulence; but which does not affect other ulcers of a specific character, such as the venereal, scrophulous, and variolous.

"Dr. Rollo has endeavoured to destroy this morbid poison by chemical means, locally applied; for this purpose he made use of the oxygenated muriatic acid, the nitrates of silver and mercury, and the oxygenated muriatic acid gas. The manner of applying these substances is as follows: —the wound ought first to be washed with luke-warm water; immediately after which the ulcerated part should be touched with the nitrat of silver; the

Printed for R. Phillips No. 71. St. Paul's Church Yard.

the wound then must be moistened with a solution of the nitrat of mercury diluted with water, or a mixture of oygenated muriatic acid and distilled water; after which, the whole is to be covered with linen dipt in one or the other of these liquids. The oxygenated muriatic gas is immediately directed to the ulcer, and the diluted solution of the nitrat of mercury is, as before prescribed, applied to the wound. By this treatment the wound soon becomes cicatrized:—this experiment has failed only in cases where the ulceration was so extensive that it could not be entirely covered with the nitrat of silver, or the oxygenated muriatic acid gas.

"One of these ulcers, after having been dusted with a considerable quantity of the nitrate of silver reduced to a fine powder, appeared to be in an excellent condition, twelve hours after this dressing; it was firm, and apparently much revived; an effect which must have been produced by the sulphurated hydrogen gas acting on the surface of the ulcer."

Dr. Rollo gives an account of some experiments published by Dr. Crawford, in the 80th volume of the "*Philosophical Transactions*" on the *matter of cancer*; and expresses his regret, that this valuable paper is not more generally known: as, says he, "*there can be little doubt of chemical changes and combinations being produced on sores.*" Dr. Rollo farther observes, "that this subject has not been carried on, since then, as it ought to have been; trusting it will gradually appear of great importance, especially as the discovery of the changes the discharge of a sore undergoes, will probably point out, at the same time, the remedy for the sore."—*A Treatise on the Diabetes Mellitus:* Edit. first. Vol. II. p. 268.

Before, however, we can proceed to give the result of Dr. Crawford's ingenious experiments, together with the opinions of this eminent medical philosopher, we shall previously furnish the reader with a concise explanation of the plate mentioned in the beginning of this paper, and which has been misplaced to our preceding Number.

*Description of the plate affixed to No. IV.*

A, (Fig. 3) is a glass bottle, or decanter, designed to contain the materials necessary to produce the gas. B, a vial, or small flask, intended to contain the acid, and which allows it to pass, at pleasure, into the decanter A, by means of the cock C.

D, is a glass-tube which serves to conduct the gas, and to the extremity of which, marked E, a bladder must be fixed. Of the smaller spherical glass vessel, with a straight and curvated tube, we find no explanation in the "*Annales de Chimie*;" and although it is there stated, that Mr. Blades, of of Ludgate-Hill, London, ſells this apparatus, yet, upon application at the warehouſe we learnt, that the latter veſſel does not form a part of his contrivance.

The bladder is uſed only, when the gas is to be applied for a certain length of time, and in order to keep it uniformly diſtended with facility, by renewing the effervescence as occaſion may require. As to the oxygenated muriatic acid gas, its effect is very quick, or almoſt inſtantaneous, ſo that it is only neceſſary to apply the orifice of the tube to the wound, for a few ſeconds.

---

Dr. Crawford concludes his experimental inquiry into the nature of cancerous matter, with the following important obſervations:

"It appears from the experiments which have been recited, that in cancerous and other malignant ulcers, the animal fibres undergo nearly the ſame changes which are produced in them by deſtructive diſtillation. The purulent matter prepared for the purpoſe of healing the ulcer is, in ſuch caſes, mixed with animal air and volatile alkali. The compound formed by the union of theſe ſubſtances, which may perhaps not improperly be termed hepatiſed ammonia, decompoſes metallic ſalts, and acts upon metals; for we have ſeen that when it was placed in a jar over mercury for ſeveral days, the ſurface of the mercury acquired a black colour, and that it inſtantly occaſioned a dark precipitate in a ſolution of nitrated ſilver. Theſe facts ſeem to afford an explanation of the changes produced in metallic ſalts, when they are applied to malignant ulcers. The volatile alkali combines with the acid of the metallic ſalt, and the animal hepatic air revives the metal, either by imparting to it the inflammable principle, or by uniting with the pure air which the ſalt is ſuppoſed to contain. The metal, thus revived, is probably in ſome caſes again corroded by the hepatiſed ammonia, which communicates to it a black colour. Thus we may account for the dark incruſtation frequently formed upon the tongue and internal fauces, when venereal ulcers of the throat are waſhed with a ſolution of corroſive ſublimate. And hence alſo the dark tinge which is frequently communicated by ill-conditioned ulcers to poultices made with a ſolution of ſugar of lead. The action of the hepatiſed ammonia likewiſe explains the reaſon why the probes are frequently corroded when they are introduced into ſinuous ulcers, or applied to the ſurfaces of carious bones. To the ſame cauſe it is probably owing, that poliſhed metallic veſſels are quickly tarniſhed when they are expoſed to the effluvia of putrid animal ſubſtances.

"From the foregoing experiments it moreover appears, that animal hepatic air imparts to the fat of animals recently killed a green colour; that it renders the muſcular fibres ſoft and flaccid, and increaſes the tendency to putrefaction. It is therefore a ſeptic principle; and hence it is extremely probable; probable, that the compound of this fluid with volatile alkali, which is found in the matter difcharged by the open cancer, produces deleterious effects: for although the mifchief in cancerous ulcers feems principally to depend on a morbid action of the veffels, whence the unhealthy ftate of the matter difcharged by fuch ulcers is fuppofed to derive its origin, yet, from the corrofion of the larger blood veffels, and the obftruction in the contiguous glands, there can be little doubt that this matter aggravates the difeafe. The experiments recited above appear to prove, that the hepatifed ammonia is the ingredient which communicates to the cancerous matter its putrid fmell, its greater thinnefs, and in a word, all the peculiar properties by which it differs from healthy pus.

"From thefe confiderations it was inferred, that a medicine which would decompofe the hepatifed ammonia, and deftroy the fetor of the animal hepatic air, without at the fame time increafing the morbid action of the veffels, would be productive of falutary effects. The nitrous acid does not deftroy the fetor of hepatic air, unlefs it be highly concentrated; and in this ftate it is well known that it fpeedily corrodes animal fubftances. But the fetor of hepatic air quickly difappears when it is mixed with the dephlogifticated marine acid, even though the latter be fo much diluted with water as to render it a very mild application. I have found that this acid, diluted with thrice its weight of water, gives but little pain when it is applied to ulcers that are not very irritable; and in feveral cafes of cancer it appeared to correct the fetor, and to produce a thicker and more healthy pus. It is proper, however, to remark, that other cafes occurred in which it did not feem to be attended with the fame falutary effects. Indeed fome cancerous ulcers are fo extremely irritable, that applications which are at all of a ftimulating nature, cannot be ventured upon with fafety. And hence, if the obfervations which I have made on the efficacy of this acid as an external application, fhould be confirmed by future experience, it muft be left to the judgment of the furgeon to determine both the degree of its dilution, and the cafes in which it may be employed with advantage.

"The dephlogifticated marine acid, as is gennerally known, has the power of deftroying the colour, the fmell, and perhaps the tafte, of the greater part of animal and vegetable fubftances. We have feen that it corrects the fetor of putrid flefh. And I have found, that when it is poured in fufficient quantity upon hemlock and opium, thefe narcotics fpeedily lofe their fenfible qualities. As it appears, therefore, to poffefs the power of correcting the vegetable, and probably many of the animal poifons, it feemed not unlikely, that it might be ufeful as an internal medicine. Conceiving that its exhibition would be perfectly fafe, I once took 20 drops of it, diluted with water. I foon afterwards, however, felt an obtufe pain, with a fenfe of conftriction in my ftomach and bowels. This uneafinefs, notwithftanding the ufe of emetics and laxatives, lafted for feveral days, and was at length removed by drinking water impregnated with sulphureous hepatic air. I afterwards found that the manganese, which had been used in the distillation of the acid, contained a small portion of lead.

"Dr. INGENHOUZ informed me, that a Dutchman of his acquaintance, some time ago, drank a considerable quantity of the dephlogisticated marine acid: the effects which it produced were so extremely violent, that he narrowly escaped with his life. If, therefore, this acid should hereafter be employed as an internal medicine, it would be necessary to prepare it by means of manganese that has been previously separated, by a chemical process, from the lead and the other metals with which that substance is usually contaminated."

This quotation has induced Dr. ROLLO to observe, that at his request, Mr. CRUIKSHANK made some experiments on the matter of this sore, and that the following account contains the result, with his remarks, as communicated to us in April 1795.

"The matter of this sore is sparingly soluble in water, but readily diffused through it, producing a milky appearance. Pure volatile alkali first reduces it to a transparent jelly, and after some time dissolves the greatest part; a similar effect is produced on pure pus. These solutions are but partially precipitated by acids, particularly the sulphuric. The tincture of litmus, and of Brazil wood are not changed by this matter; it does not therefore possess either acid or alkaline properties. If to the filtered solution of this matter in distilled water, a little nitrated silver be added, a whitish-coloured precipitate will be produced. Similar precipitates, but much more copious, are occasioned by nitrated and muriated mercury. When pure pus is treated in the same manner, these precipitates, particularly that by muriated mercury, have somewhat of a different appearance, which it would be difficult to describe. The fetid smell is somewhat changed by lime-water, but not destroyed; the sulphuric acid rather increases it; a similar effect is produced by alcohol, and by the alkaline solution of arsenic. A decoction of the Peruvian bark does not destroy the fetor. This, however, is effected by the nitrates and muriates of mercury, by the nitrous acid; but most completely by the oxygenated muriatic acid, and gas. Nitrated silver produces very little change either on its colour or smell, a circumstance the more remarkable, as this salt possesses the property of destroying most offensive smells, even that of the matter of cancer.

"It must be allowed that the offensive smell of the matter of this sore is produced by that part of the discharge which is altered from the nature of pure pus; for we know that every ill-conditioned discharge has more or less smell, while good pus has none. It is a known fact in chemistry, admitting of few exceptions, that a substance cannot have its smell totally destroyed or altered, without having its properties changed at the same time. If therefore this peculiar matter, by the addition of nitrated or muriated mercury, the oxygenated muriatic acid, &c. should have its smell completely destroyed, there is is every reason to believe that its peculiar properties also will be so; and should it be capable in its original state of producing an ill-conditioned action in sores, the addition of such substances might prevent this mischief. If it should be supposed therefore that an acrid matter somehow produced on the surface of sores, were capable of inducing ulceration of a specific kind, and that this ulceration, like the venereal, should generate more matter of a nature similar to itself, capable of extending the mischief, and even of bringing on a general affection of the system, some important conclusions might be drawn from these experiments.

"1st. It is easy to see, that a sore once clean, might be preserved from the effects of the matter alluded to, by washing it at every dressing with a weak solution of nitrated mercury, or the oxygenated muriatic acid, and that even the generation of such matter might be entirely prevented by the same means.

"2d. After the action has taken place, and before a general disposition is formed, it might be possible to put a stop to its progress by very active topical applications, such as should be capable not only of destroying the specific nature of the matter generated, but also the action itself. From the experiments already related, it is evident we would prefer in this case, the most active mercurial preparations, such as red precipitate not entirely deprived of its acid, or the muriated mercury; and if an actual caustic were to be employed, we should have recourse to the strong nitrous acid, applied in Mr. HUMPAGE's method, rather than the nitrated silver, especially as it may have also the effect of changing the nature of the discharge; this consists in dipping a little lint in the acid, and applying it to the part: it communicates less pain than any other caustic, except the nitrate of silver.

"With regard to the action of the different substances on sores, and as caustics, they may be thus arranged:

"1st. Substances exciting action, and producing death, in parts, by the excess of that action; as arsenic, and muriated mercury.

"2d. Substances acting simply by burning or destroying the part, and whose actions are always limited; as, nitrated silver, nitrated mercury, and nitrous acid.

"3d. Substances acting by dissolving the part, and whose action is so diffusive that it is difficultly limited; as, common caustic, or the mixure of potash and lime.

"4th. Substances acting chemically on the part by decomposition; as, oxygenated muriatic acid, in the form of gas, or combined with water.

"On the whole, though we have supposed the formation of a new morbid poison, on the surface of certain sores, under peculiar circumstances or management, yet we are rather inclined to change the appellation *new*, to a poison which which has been probably *overlooked*. We have feen the commencing ulceration remain fome days ftationary, we have feen it extending, while the other parts of the former fore were cicatrifing, and the conftitutional effects not taking place until the ulceration had occupied a large part of the fore; and we have feen that the painful ftate and extreme fenfibility did not occur until the fyftem was affected. Therefore it may be prefumed the early ulceration has been unattended to, and the ftate of the fore remarked only by authors after it had affumed the appearance of phagedæna. For when the ulceration had fo fpread as to produce the conftitutional affection, and the confequent rapid changes on the fore, the character of the virulent fore defcribed as phagedæna was formed.

"The account we have given of this fore may excite more attention to the ftate of a large fore in an hofpital with a confiderable difcharge, and lead to a trial of the applications pointed out; to forward cicitrifation, and prevent any untoward changes from the production of a poifon on the furface of the fore.

"Since the attention and manner of treating fores as defcribed, have been purfued in this hofpital, we have had none fuch, nor even the hofpital-fore, indeed this we cannot poffibly have, as ventilation and the deftruction of general contagion are fo carefully and unremittingly performed. We have had, however, three very remarkable fores following bubo in the groin, and chancre on the penis, which terminated fatally. Thefe cafes occurred before the adoption of the new remedies, and were treated by mercury, and appeared to be the effect of the mercurial difeafe on a peculiar conftitution. The fores were irritable and floughing, and the only favourable changes were produced by the ufe of opium, the hepatifed ammonia, and the application to the fores of the hydrogenous, hepatic, and carbonic acid gafes.

"The fore which has been defcribed and noticed by us at the beginning of this account as peculiar to hofpitals, though well marked by many, yet we have our doubts, but that many of thefe were this peculiar fore, and owing to the poifon we have fuggefted. Whatever it may be, it adds another fact in corroboration of the advantages both medicine and furgery are likely to derive from the new doctrines of chemiftry.

"We have already feen the utility of fubftances readily parting with their oxygen, applied to irritable fores, and alfo of the hydrogenous, hepatic, and carbonic acid gafes to irritable fores. See Vol. I. pag. 62, and which was contained in the Notes of the firft cafe of Diabetes, difperfed in January laft. In page 61 of the fame volume, it is obferved, that the oxygenated muriatic gas was found to deftroy the offenfive fmell of fores, that it deftroyed fpecific contagion, and could be eafily obtained, and very fafely ufed. We had therefore given it a preference to other things, and in order that it may be more generally tried, we infert Mr. Cruickfkank's manner of procuring and ufing it in the wards of this hofpital.

"This

"This confifts in intimately mixing two parts of common falt, and one of cryftallifed manganefe, previoufly reduced to powder. Two ounces of this compound are introduced into a fmall bafon; about an ounce of water is then added, and afterwards an ounce and a half of the concentrated vitriolic or fulphuric acid at different times, fo as to preferve a gradual difcharge of the oxygenated muriatic acid gas. One of thefe bafons is fufficient for a ward or room, containing five or fix beds, and more muft be employed according to the fize of the apartment."

## Figures and Tables

**Fig. 1. Fig. 2. Fig. 3. f1:**